# Sinonasal Carcinoma With Aggressive Right Intraorbital Extension and Left Eye Invasion in a Young Female Patient

**DOI:** 10.7759/cureus.74066

**Published:** 2024-11-20

**Authors:** Nur Ain Shafiyah Mohd Ghazali, Wan-Hazabbah Wan Hitam, Nur Hafizah Maffar

**Affiliations:** 1 Department of Ophthalmology and Visual Sciences, School of Medical Sciences, Universiti Sains Malaysia, Kota Bharu, MYS

**Keywords:** bilateral intraorbital extension, intracranial extension, proptosis, sinonasal carcinoma, snscc

## Abstract

Sinonasal cancers are rare and aggressive head and neck malignancies. Sinonasal squamous cell carcinoma (SNSCC) typically affects males and individuals over the age of 55. Here, we present an unusual case of a young female diagnosed with SNSCC. She presented with painful right eye proptosis, rapid progressive vision loss in both eyes, and a history of intermittent epistaxis. MRI revealed an aggressive sinonasal mass with intra-orbital and intracranial extension, and a biopsy confirmed sinonasal non-keratinizing squamous cell carcinoma. Despite initial systemic chemotherapy, the patient discontinued treatment and, unfortunately, succumbed to the disease. This case highlights the aggressive nature and management challenges of advanced SNSCC, emphasizing the critical importance of early diagnosis and timely intervention to improve outcomes.

## Introduction

Sinonasal cancers are rare head and neck malignancies, comprising less than 1% of all cancers overall [1]. They predominantly consist of tumors of epithelial origin [2], with squamous cell carcinoma being the most common histologic subtype, accounting for 60-75% of malignancies in the paranasal sinuses [3,4]. Sinonasal squamous cell carcinoma (SNSCC) predominantly affects males and patients over 55 years of age [1,3-6]. These tumors can arise anywhere within the sinonasal cavities, though they most commonly originate in the nasal cavity and maxillary sinus [3,4].

Patients with sinonasal malignancies often present with non-specific symptoms, leading to delayed diagnosis due to the proximity of critical anatomical structures within the sinonasal region. The differential diagnosis for sinonasal masses should also include conditions such as invasive fungal rhinosinusitis and inverted papilloma, which can present with similar symptoms. Clinical assessment begins with a thorough medical history, an ear, nose, and throat examination, and an evaluation of adjacent cranial nerves. Non-invasive imaging techniques such as CT and MRI are used to determine tumor location, extent, and characteristics, assisting in clinical staging and treatment planning [2].

The prognosis for patients with sinonasal carcinomas is generally unfavorable, with five-year survival rates heavily influenced by the stage at diagnosis.

This report presents a rare and dramatic case of advanced SNSCC in a young female, who exhibited the striking clinical manifestations of severe right eye proptosis and rapid, bilateral vision loss.

## Case presentation

An 18-year-old female student presented with a one-month history of progressive right eye severe non-axial proptosis, accompanied by acute vision loss over the past week. She had experienced recurrent episodes of epistaxis and bilateral nasal obstruction two months earlier, for which she received treatment for allergic rhinitis. There was no history of radiation exposure or smoking, and her family history was unremarkable.

On examination, she exhibited right-sided facial fullness and non-axial proptosis in the right eye. Extraocular movements were restricted in all directions. There was no light perception in the right eye, while visual acuity in the left eye was preserved at 20/20. Slit lamp examination revealed conjunctival chemosis and exposure keratitis secondary to severe lagophthalmos. The lens and vitreous in both eyes were clear. Fundoscopy of the right eye revealed optic disc swelling. The anterior and posterior segments of the left eye were normal. Intraocular pressure measurements were within normal limits. Hertel's exophthalmometry confirmed right eye proptosis, with measurements of 27 mm OD and 17 mm OS. Neurological examination revealed multiple cranial nerve deficits, affecting the right cranial nerves II, III, IV, and VI, as well as bilateral olfactory nerve impairment. There was also reduced sensation in the ophthalmic and maxillary divisions of the right trigeminal nerve.

**Figure 1:**
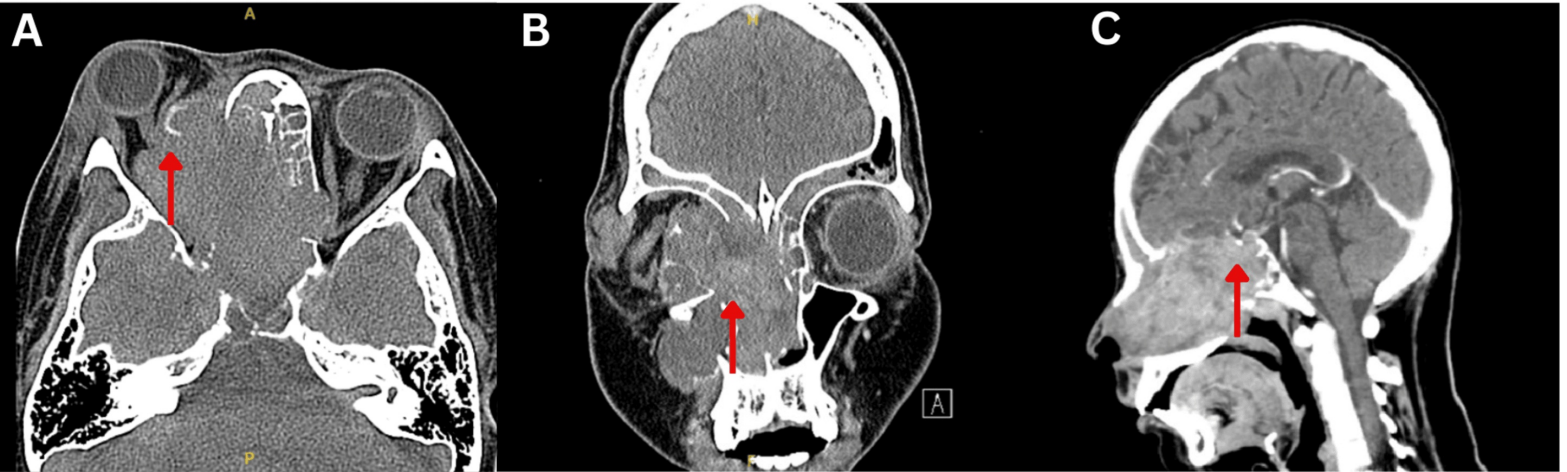
Contrast-enhanced computed tomography of the brain and paranasal sinus. (A) Axial CT showed a large, heterogeneously enhancing lobulated solid mass occupying the right nasal cavity and paranasal sinuses. It extends into the medial aspect of the right intra- and extraconal fat spaces, causing right proptosis and lateral displacement of the right optic nerve (arrow). Poor fat plane of the right medial rectus muscle. (B) Coronal view revealed bony erosion of the nasal septum, right maxillary sinus walls, and both ethmoid air cells. A large mass (arrow) occupies the right nasal cavity extending into the right orbit and surrounding paranasal sinus. (C) Sagittal view showed an extension of the mass (arrow) into the sphenoid sinus and pituitary fossa with the erosion of the planum sphenoidale, sella turcica, and clivus.

A contrast-enhanced computed tomography (CECT) scan of the brain, orbits, and paranasal sinuses demonstrated a large, heterogeneously enhancing, lobulated mass occupying the right nasal cavity and paranasal sinuses, with erosion of the nasal septum, right maxillary and sphenoid sinus walls, and both ethmoid air cells. These findings were suggestive of an aggressive sinonasal tumor with locoregional extension (Figure 1).

**Figure 2:**
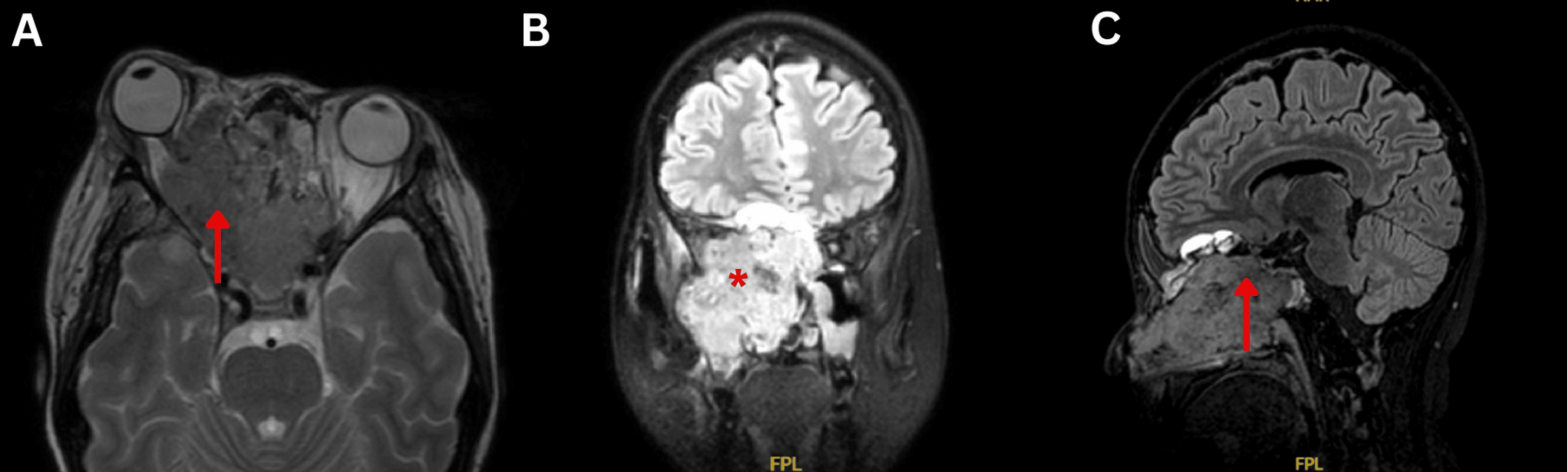
Magnetic resonance imaging of the brain and orbit. (A) Axial T2-weighted MRI showed a massive, well-defined, heterogeneous sinonasal mass extended into bilateral orbits, with greater right orbit involvement (arrow). (B) Coronal T2-weighted MRI showed the mass epi-centred at the right maxillary, sphenoid, ethmoid, and nasal cavity (asterisk). (C) Sagittal fluid-attenuated inversion recovery (FLAIR) MRI showed extension (arrow) of the sinonasal mass into the anterior cranial fossa.

Magnetic resonance imaging (MRI) of the brain and orbits, with and without gadolinium, revealed a well-defined heterogeneous sinonasal mass measuring 4.4 cm × 5 cm × 6.1 cm, with intracranial and orbital extension, right orbital infiltration, extraocular muscle displacement, and optic nerve encasement (Figure 2).

**Figure 3:**
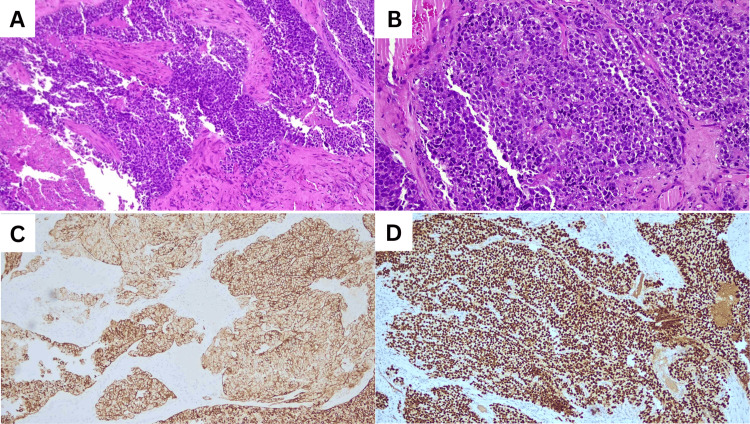
Histopathology and immunohistochemistry assessment of the mass. (A) The section demonstrates malignant epithelial cells arranged in sheets and nests, infiltrating the stroma (haematoxylin and eosin (H&E), 100x magnification). (B) The malignant epithelial cells display moderate pleomorphism, featuring round to enlarged nuclei, some with prominent nucleoli, and moderate pale to eosinophilic cytoplasm. Keratin pearls and intercellular bridges are absent (hematoxylin and eosin (H&E), 200x magnification). (C) The malignant epithelial cells are positive for CKAE1/AE3, epithelial marker (immunohistochemistry (IHC), 100x magnification). (D) The malignant epithelial cells exhibit nuclear positivity for p63, a squamous epithelial marker, as shown by immunohistochemistry (IHC) at 100x magnification.

The patient was referred to otorhinolaryngology, where a nasopharyngoscopy revealed an ulcerated mass occupying the right nasal cavity with a significant loss of anatomical structure. The biopsy confirmed a diagnosis of non-keratinizing squamous cell carcinoma, with immunohistochemistry showing positive staining for CK AE1/AE3, p63, and CK 5/6 (Figure 3). A diagnosis of advanced sinonasal non-keratinizing squamous cell carcinoma with intraorbital and intracranial extension was made.

The patient was scheduled for three cycles of systemic induction chemotherapy at three-week intervals. This is followed by either surgical surgery or adjuvant concurrent chemoradiotherapy, contingent upon the response. She underwent only two cycles of intravenous chemotherapy, consisting of docetaxel 60 mg/m^2 ^and cisplatin 60 mg/m^2^ on day one, and fluorouracil 750 mg/m^2^, on day one to day five. She was administered pain treatment with oral morphine 10 mg, at four-hour intervals. She also had intensive topical antibiotics, comprising guttae ceftazidime 5% and guttae moxifloxacin 5%, along with intensive topical lubricants. However, the patient failed to attend further follow-up visits after being discharged home.

One month later, she presented at the emergency department with an intense headache and abrupt visual loss in the left eye. She had inadequate oral intake and lethargy. Clinical examination (Table 1) revealed a larger tumor extending to both nostrils and oral region. It was accompanied by discomfort and contact bleeding. The fundoscopic evaluation of the left eye demonstrated edema of the optic disc. A subsequent CECT brain scan revealed a growing mass, indicating an enhanced mass effect. She was admitted to the intensive care unit due to sepsis secondary to an infected tumor, accompanied by severe anemia. Her full blood count showed a leukocytosis of 14.7 x 10^9^/L and a hemoglobin level of 4.7 g/dL. However, she declined further diagnostic blood work. She was started on intravenous Rocephin at a dose of 1 g daily and received a transfusion of two units of red blood cells. Despite these interventions, she ultimately succumbed to her illness on the third day of hospitalization.

**Table 1:** Summary of the clinical examination of the patient at presentation. NLP = no light perception; CN = cranial nerve

Examination	Right Eye	Left Eye
Vision	NLP	20/20
External Ocular Examination		
Eyeball	Non-axial proptosis	Normal
Extraocular Movement	Restricted all gaze	Full
Exophthalmometer (mm)	27	17
Slit Lamp Examination		
Conjunctiva	Injected prolapsed conjunctiva with chemosis	White
Cornea	Inferior half of cornea epithelial defect	Clear
Lens	Clear	Clear
Vitreous	Clear	Clear
Funduscopy	Optic disc swelling	Normal optic disc
IOP (mmHg)	12	13
Other Examination		
Cranial Nerve	Impairment of CN I, III, IV, VI	Impairment of CN I

## Discussion

SNSCC is an uncommon and aggressive tumor, predominantly located in the maxillary sinus or nasal cavity [4-6]. It generally manifests in the sixth and seventh decades of life, with a male-to-female ratio 2:1 [1,4,6]. The rarity of SNSCC in young adult populations underscores the importance of investigating this diagnosis, especially in patients with atypical symptoms such as proptosis and visual impairment. This instance highlights doctors' need to remain vigilant when assessing cancers in younger patients who may not fit conventional risk profiles.

Table 2 highlights the clinical appearance, staging, and treatment options for sinonasal cancer as reported in previous publications.

**Table 2:** Clinical presentation, disease staging, and treatment modalities of sinonasal carcinoma in the literature.

Aspect	Details	References
Presenting Features	Most common nasal obstruction. Others: Epistaxis, facial pain/swelling, swollen eye. These symptoms can delay diagnosis as they mimic benign sinus conditions.	Mahalingappa et al. (2014), Yee et al. (2024,) Haque et al. (2014)
Staging of Disease American Joint Committee on Cancer (AJCC)	TNM Staging, refer to Table 3	Doescher et al. (2017)
Treatment Modalities	Surgery: Endoscopic or open resection is preferred, especially for accessible tumors. Radiotherapy: Post-operative therapy for local control, or as primary therapy for unresectable cases. Chemotherapy: Adjuvant therapy for advanced disease, often cisplatin-based regimens. Immunotherapy: Currently experimental, being explored for recurrent or metastatic SNC cases.	Hanna et al. (2011), Robin et al. (2017)

The commonly described presenting symptoms in the literature include nasal blockage, epistaxis, rhinorrhea, and facial pain [4,5]. Mahalingga et al. and Haque et al. similarly identified nasal blockage as the most common presentation in their studies [7,8]. Proptosis (bulging of the eyes), diplopia (double vision), or neurological symptoms can be present in patients with advanced-stage tumors [2]. Our patient displayed symptoms such as proptosis, visual impairment, and nasal obstruction, indicating locally advanced disease. The swift advancement of her illness underscores the aggressive characteristics of SNSCC, frequently resulting in considerable morbidity and management difficulties [1,3,9].

Imaging tests are essential for treatment planning and identifying the most effective surgical intervention or radiation therapy methods. Sinonasal carcinoma typically presents as a soft tissue mass in the sinonasal region, often leading to sinus opacification and mucosal thickening. CT scans may reveal bone invasion and enlarged cervical lymph nodes, while MRI shows intermediate to low signal on T1 and high signal on T2-weighted images, with heterogeneous enhancement post-contrast [10,11]. CECT of this patient demonstrated a substantial, lobulated sinonasal mass within the right nasal cavity, extending into the paranasal sinuses, accompanied by erosion of neighboring anatomical structures. The MRI of this patient revealed intracranial and orbital extension, displacement of extraocular muscles, and encasement of the optic nerve.

The staging of sinonasal malignancies follows the American Joint Committee on Cancer (AJCC) tumor/node/metastasis (TNM) classifications for cancers of the nasal cavity and paranasal sinuses [12]. Table 3 delineates the TNM staging of sinonasal cancers.

**Table 3:** Tumor/node/metastasis (TNM) staging of sinonasal malignancies according to the American Joint Committee on Cancer AJCC Cancer Staging Manual, Eighth Edition.

TNM Staging	
T Stage	
Tis	Carcinoma in situ
T1	Tumor limited to one subsite (septum, floor, lateral wall, or vestibule [edge of naris to mucocutaneous junction of nasal cavity]; left or right ethmoid sinus)
T2	Tumor involving two subsites in one region or extending to involve an adjacent region in the nasoethmoidal complex
T3	Tumor invades any of the following: • Medial wall or floor of orbit • Maxillary sinus • Palate • Cribriform plate
T4a	Tumor invades any of the following: • Anterior orbital contents • Skin of nose or cheek • Minimal extension to anterior cranial fossa • Pterygoid plates • Sphenoid sinus • Frontal sinus
T4b	Tumor invades any of the following: • Orbital apex • Middle cranial fossa • Dura • Brain • Cranial nerves other than maxillary division of trigeminal nerve (V2) • Nasopharynx • Clivus
N stage	
N0	No regional node metastases
N1	Metastasis in single ipsilateral node, ≤3 cm, and no extranodal extension (ENE−)
N2a	Metastasis in single ipsilateral node, >3 and ≤6 cm, and ENE−; or metastasis in single ipsilateral node, ≤3 cm, and ENE-positive (ENE+)
N2b	Metastasis in multiple ipsilateral nodes, all ≤6 cm, and ENE−
N2c	Metastasis in bilateral or contralateral nodes, all ≤6 cm, and ENE−
N3a	Metastasis in a node, >6 cm, and ENE−
N3b	Metastasis in single ipsilateral node, >3 cm, and ENE+; or multiple ipsilateral, contralateral, or bilateral nodes any with ENE+; or single contralateral node of any size and ENE+

Histological assessment is crucial for diagnosis, prognostication, and informing therapy choices. Histological examination validated the diagnosis of non-keratinizing squamous cell carcinoma. Immunohistochemical staining of this patient revealed positive for CK AE1/AE3, p63, and CK 5/6, which are indicative markers of squamous cell carcinoma. The existence of these markers aids in differentiating SNSCC from other sinonasal neoplasms and verifying its epithelial origin [9,13].

Human papillomavirus (HPV) immunohistochemistry is often recommended in the evaluation of sinonasal carcinoma in young, non-smoking patients due to its association with HPV-related carcinogenesis. HPV-positive sinonasal carcinomas tend to have distinct histopathological and molecular profiles, which can impact prognosis and therapeutic approaches [14]. However, in this particular case, HPV immunohistochemistry was not performed, limiting the potential for HPV status to contribute to the diagnostic or prognostic assessment.

A thorough differential diagnosis must be excluded when assessing sinonasal masses, particularly in younger patients. This encompasses benign tumors such as inverted papilloma or nasopharyngeal angiofibromas, which are more prevalent in adolescent boys and typically exhibit a more favorable prognosis. Malignant neoplasms, including adenocarcinomas, lymphomas, olfactory neuroblastomas, sinonasal undifferentiated carcinoma, and nasopharyngeal carcinomas, must also be considered. Sinus infections, particularly fungal infections, can lead to considerable nasal blockage and proptosis in immunocompromised individuals, necessitating thorough evaluation to rule out malignancies [9].

The treatment for sinonasal carcinomas involves surgery for resectable tumors, which may be performed before or after chemotherapy and radiation therapy in locally advanced cases. Patients who received adjuvant radiotherapy, adjuvant chemoradiotherapy, or neoadjuvant therapy had improved overall survival compared to those who received surgery alone as reported by Robin et al. [15]. Hanna et al. documented a two-year overall survival (OS) rate of 77% for patients with locally advanced SNSCC who underwent induction therapy before definitive treatment [16].

**Figure 4:**
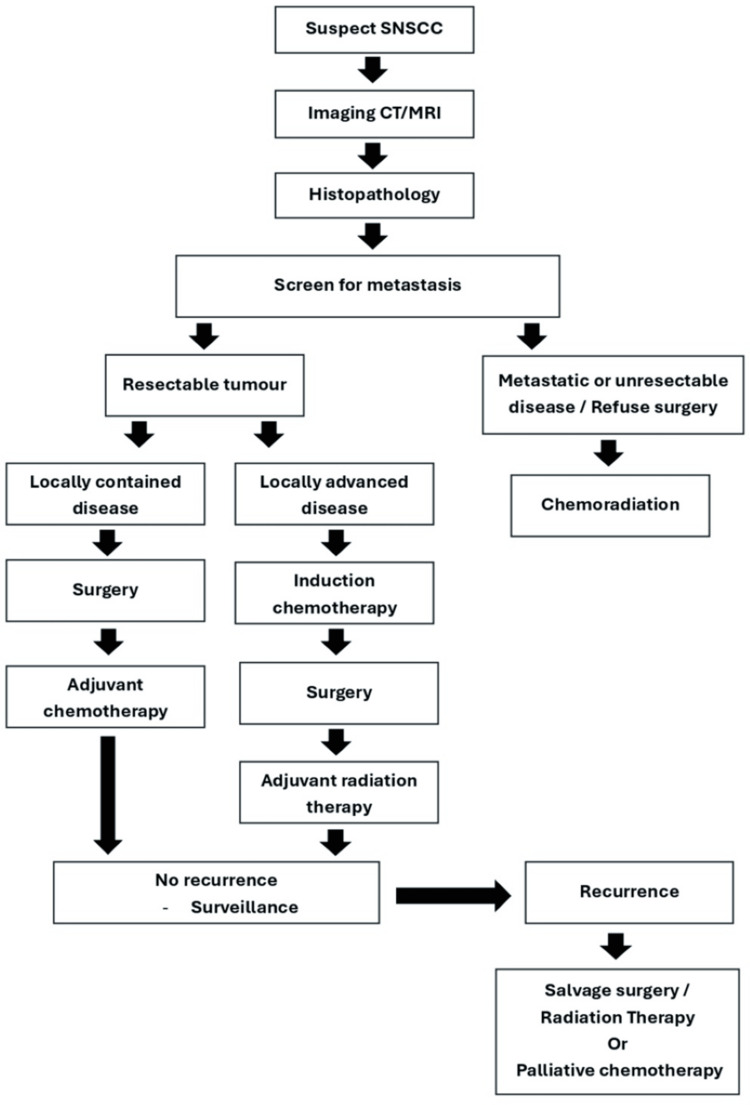
Summary of the management of sinonasal carcinoma. SNSCC = sinonasal squamous cell carcinoma; CT = computed tomography; MRI = magnetic resonance imaging

For unresectable tumors and for those who do not choose to undergo surgery, chemoradiation - typically using intensity-modulated radiation therapy (IMRT) and platinum-based chemotherapy - is the standard approach. In cases of recurrence, salvage surgery or re-irradiation is preferred, with palliative chemotherapy as an option if surgery is not feasible. Advanced or metastatic cases primarily rely on systemic chemotherapy, with immunotherapy considered in select cases. Regular post-treatment monitoring is essential due to the high risk of recurrence [9,13,17,18]. In our patient's case, she was initiated on systemic chemotherapy but neglected follow-up treatment. This oversight led to rapid disease progression and deterioration due to complications, including a superimposed infection, highlighting the complexities of managing this condition. Figure 4 summarizes the management of sinonasal carcinoma.

Sanghvi et al. identified variations in survival rates based on disease progression, noting that individuals with distant metastases had the lowest survival rates [1]. This observation aligns with earlier findings, as squamous cell carcinoma of the paranasal sinuses is notoriously difficult to diagnose due to the typically asymptomatic nature of the tumors [8]. Published five-year survival rates for these advanced malignancies are approximately 50% [1,8]. The patient's subsequent death due to complications underscores the poor prognosis often associated with advanced sinonasal carcinoma.

SNSCC is a rare and aggressive tumor that often presents with atypical symptoms in younger patients, highlighting the importance of vigilance and thorough investigation in this demographic. Accurate diagnosis and effective treatment planning rely heavily on comprehensive imaging and histological assessment, as specific markers identified through immunohistochemical staining can help differentiate SNSCC from other sinonasal neoplasms.

## Conclusions

This case of advanced SNSCC in a young adult underscores the rarity of this disease, with an incidence of only 0.32 cases per 100,000 people. It also highlights the aggressive nature of a malignancy that is typically observed in older individuals. The rapid onset of symptoms, including proptosis and vision loss, underscores the need for prompt diagnosis and treatment. The patient's death, despite initial treatment, illustrates the challenges of managing advanced sinonasal cancers, particularly due to their complex anatomy and rapid progression. This case emphasizes the importance of heightened awareness and timely intervention by healthcare providers to improve outcomes for affected patients.
